# Design and Modelling of a Continuum Robot for Distal Lung Sampling in Mechanically Ventilated Patients in Critical Care

**DOI:** 10.3389/frobt.2021.611866

**Published:** 2021-05-03

**Authors:** Zisos Mitros, Balint Thamo, Christos Bergeles, Lyndon da Cruz, Kevin Dhaliwal, Mohsen Khadem

**Affiliations:** ^1^Robotics and Vision in Medicine (RViM) Lab, School of Biomedical Engineering & Imaging Sciences, King's College London, London, United Kingdom; ^2^Wellcome/EPSRC Centre for Interventional and Surgical Sciences, University College London, London, United Kingdom; ^3^School of Informatics, University of Edinburgh, Edinburgh, United Kingdom; ^4^Translational Healthcare Technologies Group in the Centre for Inflammation Research, Queen's Medical Research Institute, Edinburgh, United Kingdom

**Keywords:** surgical robot, robotic bronchoscope, mathematical modelling, steerable catheter, flexible robot

## Abstract

In this paper, we design and develop a novel robotic bronchoscope for sampling of the distal lung in mechanically-ventilated (MV) patients in critical care units. Despite the high cost and attributable morbidity and mortality of MV patients with pneumonia which approaches 40%, sampling of the distal lung in MV patients suffering from range of lung diseases such as Covid-19 is not standardised, lacks reproducibility and requires expert operators. We propose a robotic bronchoscope that enables repeatable sampling and guidance to distal lung pathologies by overcoming significant challenges that are encountered whilst performing bronchoscopy in MV patients, namely, limited dexterity, large size of the bronchoscope obstructing ventilation, and poor anatomical registration. We have developed a robotic bronchoscope with 7 Degrees of Freedom (DoFs), an outer diameter of 4.5 mm and inner working channel of 2 mm. The prototype is a push/pull actuated continuum robot capable of dexterous manipulation inside the lung and visualisation/sampling of the distal airways. A prototype of the robot is engineered and a mechanics-based model of the robotic bronchoscope is developed. Furthermore, we develop a novel numerical solver that improves the computational efficiency of the model and facilitates the deployment of the robot. Experiments are performed to verify the design and evaluate accuracy and computational cost of the model. Results demonstrate that the model can predict the shape of the robot in <0.011s with a mean error of 1.76 cm, enabling the future deployment of a robotic bronchoscope in MV patients.

## 1. Introduction

Critically ill patients who develop respiratory failure and require mechanical ventilation (MV) suffer a high morbidity and mortality. Indeed, Covid-19 patients who require MV, have a mortality approaching 40% in some case series. Once MV, patients are at high risk of developing secondary infections and other secondary complications. Rapid and accurate sampling of the distal lung is an important diagnostic procedure to guide therapeutic interventions. However, despite the high cost and attributable morbidity and mortality, diagnosis of diseases in the distal lung in MV patients is not standardised, lacks reproducibility and requires expert operators. Often, this leads to empirical treatments such as broad spectrum antibiotics which are then very difficult to deescalate, thus compounding the exposure of patients to non-targeted antimicrobials and promoting antimicrobial resistance. Pulmonary infiltrates in MV critically ill patients are a common occurrence and a major diagnostic challenge. Endobronchial secretions such as mucus and often hinder manually steered bronchoscopes, leading to poor sampling results. Hence, the aim of this paper is to develop a robotic bronchoscope that democratises sampling of the lung in MV ICU patients and enables non-skilled operators to safely sample disparate regions of the human lung to improve diagnostic accuracy and therapeutic interventions.

**Figure 1:**
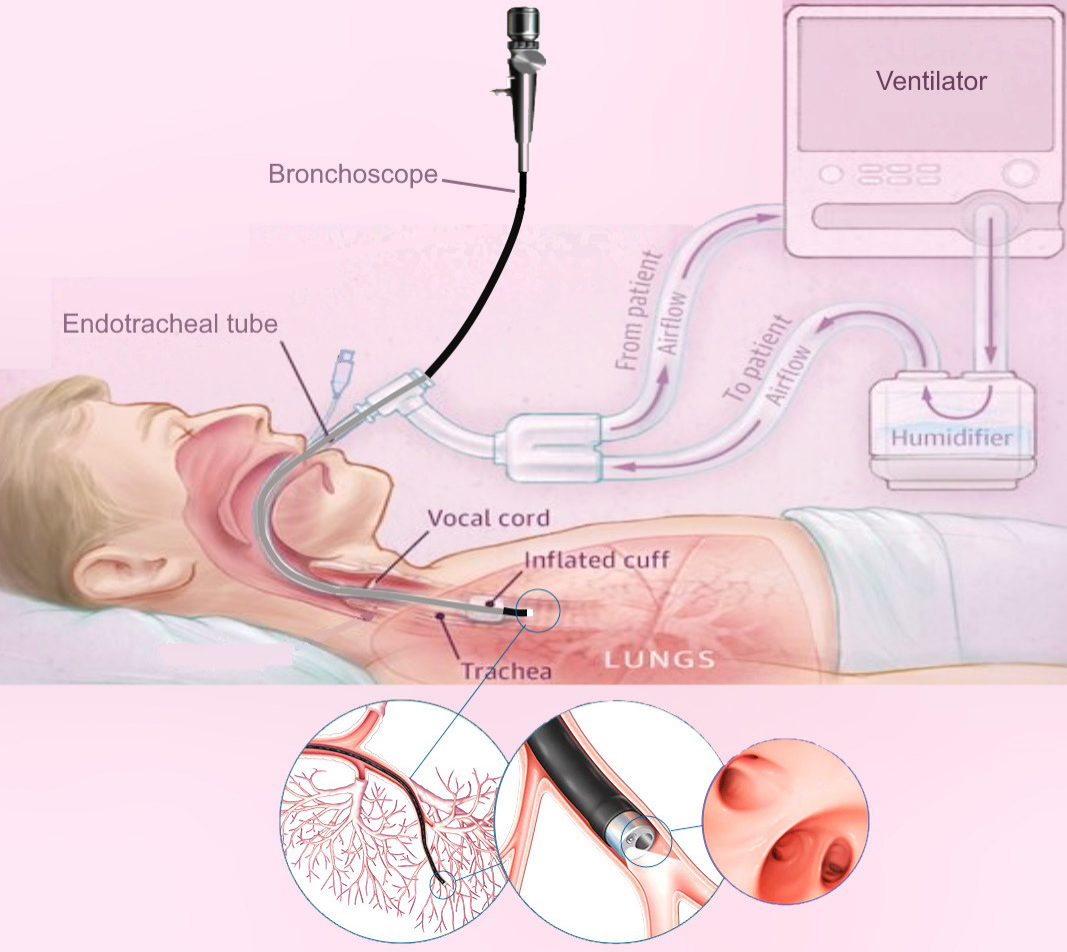
A schematic of lung bronchoscopy in ICU, showcasing the insertion of the robotic bronchoscope through the mechanical ventilator and inside the lung.

Bronchoscopy is a common diagnostic modality for the early detection of lung diseases (see Figure 1). During bronchoscopy, a thin tube (bronchoscope) is passed through the vocal cords into the airways to reach potential regions of the lung for directed sampling. Due to relatively large dimensions of the bronchoscope used for sampling (> 5 mm), bronchoscopy of MV patients is challenging. Another major drawback of the current technology is reliance on manual insertion, which is difficult due to the limited Degrees of Freedom (DoFs) of the bronchoscope, i.e., rotation and insertion.

To address the aforementioned challenges, we have developed a miniaturised continuum robot for lung bronchoscopy. A continuum robot has a continuously elastic structure and can traverse tightly curved 3D paths in confined spaces and reach desired positions deep inside human cavities. Continuum robots retain force transmission capability and offer great dexterity, thus, enabling optimal therapies when deeply seated pathologies are targeted (Burgner-Kahrs et al., 2015). Continuum robots have been explored for various interventions including laparoscopy (Wu et al., 2019), cardiac surgery (Fagogenis et al., 2019), neuro-surgery (Mattei et al., 2014), and eye surgery (Mitros et al., 2020).

The proposed bronchoscope is a continuum robot comprised of several parallel rods that can be bent via pushing/pulling of the rods. A continuum robot composed of several constrained push/pull rods is commonly known as a *multi-backbone robot*, first introduced in Gravagne and Walker (2000). Simaan et al. introduced the first surgical multi-backbone robot for dexterous tool manipulation in robotics surgery (Simaan et al., 2004; Ding et al., 2013). Xu et al. (2015) improved this design using a “dual continuum” actuation mechanism that increases modularity. Several researchers have explored the possibility of using a parallel multi-backbone approach without constraints, allowing more dexterous robots with increased DIFs per section (Bryson and Rucker, 2014; Wang et al., 2019). Multi-backbone robots have been commonly proposed for abdominal surgeries (Garbin et al., 2019; Riojas et al., 2019; Wu et al., 2019).

A major challenge in deployment of miniaturised continuum robots is real-time and precise modelling. There are several different kinematic and dynamic models presented in the literature (see Webster and Jones, 2010; Burgner-Kahrs et al., 2015 for a detailed review). The most common model for multi-backbone robots is a geometric model proposed in Simaan et al. (2004). The model has been used to control the motion of the robot as well as contact forces at the robot's tip (Bajo and Simaan, 2016). The geometric model assumes the robot curvature is constant and provides an accurate description of the robot's differential kinematics for large scale movements. However, due to the effects of unknown boundary conditions and the constant curvature assumption, the model's prediction of the robot shape and micro-scale movements are not accurate. To overcome this challenge, Del Giudice et al. (2017) proposed a method to improve micro-scale motion of a multi-backbone robot using modulation of the flexural rigidity of the rods. Another commonly method for modelling of multi-backbone robots is Cosserat rod theory (Bryson and Rucker, 2014; Wang et al., 2019). However, the Cosserat rod theory results in a relatively large boundary value problem (BVP) that should be solved for every rod in the robot and are computationally expensive. As a result, less accurate modelling methods are still attractive due to their low computational cost (Kaouk et al., 2014; Bajo and Simaan, 2016).

In this paper, we develop a bronchoscope using a miniaturised multi-backbone robot. The bronchoscope is mounted on a linear stage that can be used to automatically insert/retract the bronchoscope to reach targeted positions in the distal lung. Next, we develop a geometrically exact model of the robot that considers both the geometry of robot and mechanical properties of the backbones. The model results in a reduced order BVP and can be used to predict the shape of the bronchoscope without the constant curvature assumption. Furthermore, we develop a novel nonlinear observer that significantly improves the computational efficiency of the model to estimate the solution of the proposed model in real-time. Finally, simulations and experiments are performed to validate the design and the modelling Theory.

In the next section, section 2.1, the robot architecture and bronchoscope design is presented. Section 2.2 details the model of the bronchoscope. Section 2.4 outlines the detail of the observer design. In section 3, simulations and experimental results are performed to evaluate the design and quantify the accuracy and computational efficiency of the model. Concluding remarks appear in section 4.

## 2. Materials and Methods

### 2.1. System Design and Prototyping

**Figure 2:**
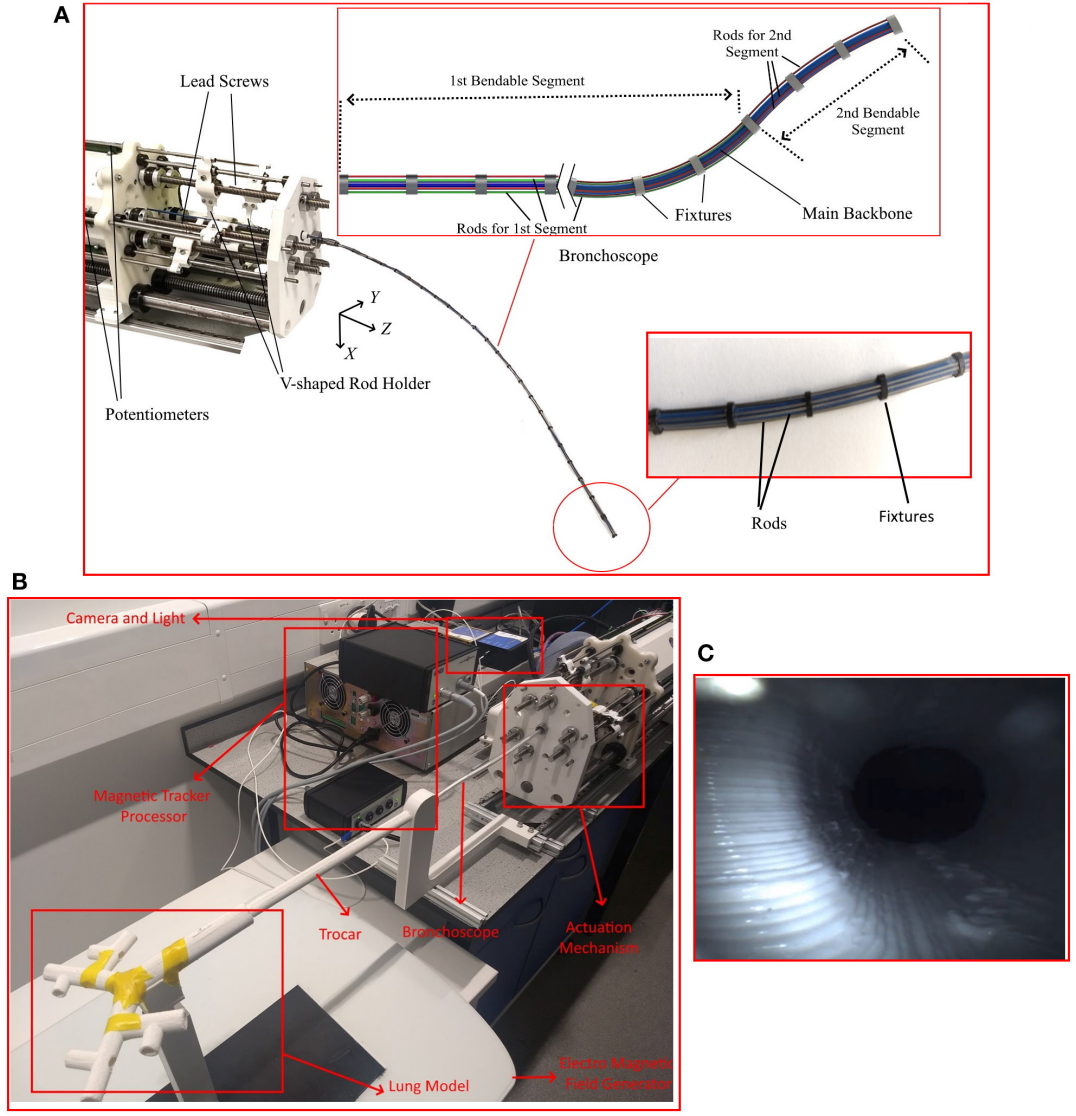
The robotic bronchoscope. **(A)** The inlet shows the tip of the bronchoscope which is composed of two segments that can be independently bent. By pulling/pushing the wires at each segment the bronchoscope can bend in 3D space. **(B)** The bronchoscope prototype placed inside a 3D printed lung model. An electromagnetic tracker (Aurora electromagnetic tracking system, NDI, Canada) is placed at the tip of the bronchoscope to measure its tip position in real-time. **(C)** Camera view from the endoscopic camera placed inside the working channel of the bronchoscope.

This section describes the design and engineering of the robotic bronchoscope. The mechanical system design begins with DoFs discussion. To improve the dexterity of the bronchoscope, we propose a novel design that allows the robotic bronchoscope to bend in 3D at two points. The tip of the bronchoscope is composed of two segments shown in Figure 2. Each segment is actuated by 3 nitinol (NiTi) rods with an outer diameter of 0.475 mm which are passed through holes located on fixtures surrounding the bronchoscope. The circular fixtures are employed to avoid buckling of the rods. An additional silicone rod shown in blue in Figure 2 is acting as the main backbone. It has an outer and inner diameters of 2.3 mm and 2 mm, respectively and is rigidly connected to the fixtures to ensure they cannot move relative to each other. The fixtures' outer and inner diameters are 4.5 and 2.4 mm, respectively. Length of proximal segment at the tip of the bronchoscope is 40 mm, length of the distal segment is 500 mm, and the overall length of the bronchoscope is 540 mm. The end-effector is actuated via the push-pull of the 6 rods. In contrast to the cable driven bronchoscopes, the proposed design employs in-compressible Nitinol rods to offer more bending curvature via pushing of the rods. Furthermore, a 7th DoFs is employed for the insertion and retraction of the end-effector into the airways.

All DoFs are actuated by brushless DC motors (Maxon Motors) with a gearhead with a 150:1 reduction and a quadratic encoder. Each motor is controlled via a position controller module with a built in PID controller (EPOS4 Compact 50/5 CAN). The controllers employ the encoders feedback to accurately control the position of the motor shaft. The position controllers communicate with a PC via the CAN protocol. A CAN-to-USB interface (Kvaser Inc., CA, USA) is used to connect the position controllers to the PC.

The motors are connected to lead screws that convert the power generated by the motor into feed velocity for pulling/pushing the rods. The lead screws are carrying a v-shaped 3D printed part that is connected to the rods (shown in Figure 2) and travels along the lead screw to pull/push the rods. Additionally, 6 linear potentiometers are used to accurately measure the displacement of the rods.

Figure 2 shows the developed robot and an inlet showcases the different segments that the manipulator comprises.

### 2.2. Geometrically Exact Model of the Robot

We use the Cosserat-rod theory (Antman, 2005; Rucker et al., 2010) to model the robot. First, we present the model for a robot with one bendable segment. Next, we generalise the model to a robot with more segments. The following notation is used throughout the paper: *x*, *x*, and **X** denote a scalar, a vector, and a matrix, respectively. A complete summary of variables and operators is given in the Appendix. The symbols used are summarised in a nomenclature section.

**Figure 3:**
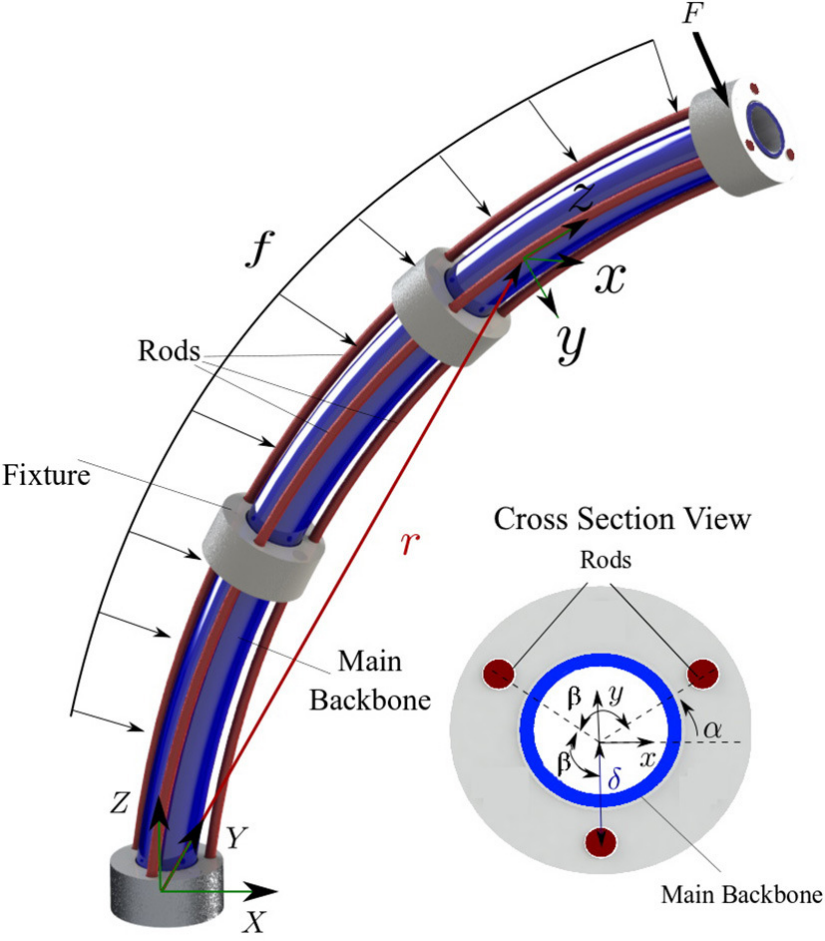
A schematic of the continuum robot with one bent segment. The main backbone is modelled as a Cosserat rod under external point force (*F*) and distributed load (*f*). The cross section view shows the position of the rods with respect to the main backbone.

A schematic of the robot is shown in Figure 3. The robot comprises a main backbone (shown in blue) rigidly connected to the fixtures and three NiTi rods (shown in red) fixed at the end fixture. The three rods can pass through the rest of the fixtures and have enough clearance to not create forces and moments but rather follow the curvature of the main backbone. The relative position of each rod with respect to the main backbone (***d***_*i*_, *i* = 1, 2, 3 in Figure 3) is given by

(1) $$\begin{array}{l} d_{i}={\left[\delta \text{cos}\left(\beta_{i}\right),\;\delta \text{sin}\left(\beta_{i}\right),\;0\right]}^{T}, \end{array}$$

where *δ* is the rods' distance from the robots centroid (see Figure 3) and *β*_*i*_ is the relative angular position of each rod with respect to the main backbone

(2) $$\begin{array}{l} \beta_{i}=\alpha +\left(i-1\right)\frac{2\pi }{3},\;i=1,2,3, \end{array}$$

with *α* shown in Figure 3.

The robot main backbone is modelled as a long, slender, one-dimensional Cosserat rod endowed with a Darboux frame attached to every point on its arc with the *z* axis of the frame tangent to the curve. The rod is under an external point force [*F*(*t*)] and distributed constant load (*f*) simulating the weight of the fixtures. The configuration of the rod can be defined using a unique set of 3D centroids, **r**(*s, t*) :[0, ℓ] × [0, ∞] → ℝ^3^ × [0, ∞], and a family of orthogonal transformations, **R**(*s, t*) :[0, ℓ] × [0, ∞] → *so*(3) × [0, ∞]. The position of the main backbone is defined by

(3) $$r\prime (s,t)=\text{R}(s,t)e_{3},\;{\text{R}}^{\prime }(s,t)=\text{R}(s,t){\left[\text{u}(s,t)\right]}_{\times },$$

where $u\left(s,t\right)={\left[u_{x}\left(s,t\right),u_{y}\left(s,t\right),u_{z}\left(s,t\right)\right]}^{T}$ is the curvature vector of the deformed backbone, [.]_×_ operator is the isomorphism between a vector in *ℝ*^3^ and its skew-symmetric cross product matrix, and $e_{3}={\left[0,\;0,\;1\right]}^{T}$ is the unit vector aligned with the z-axis of the global coordinate frame. Assuming the rods are made of linear elastic isotropic materials, we can derive the constitutive equations for calculating the instantaneous curvature of the rod (Rucker et al., 2010)

(4) $$\begin{array}{l} u^{\prime }(s,t)=-K^{-1}[{\left[\text{u}(s,t)\right]}_{\times }Ku(s,t)+\\ \;{\left[e_{3}\right]}_{\times }{\text{R}}^{T}(s,t)(F(t)+(l-s)f)], \end{array}$$

where *l* is the length of the main backbone and K = diag(*EI, EI, GJ*) is the stiffness matrix for the whole robot; *E* is the robot's Young's modulus; *I* is the second moment of inertia; *G* is the shear modulus; *J* is the polar moment of inertia. It is assumed that the cross section of the robot is symmetric and the products of inertia are negligible (i.e., *I*_*xy*_ = *I*_*xz*_ = *I*_*yz*_ ≃ 0)

In practice, the robot curvature ***u***(*s, t*) and position ***r***(*s, t*) are unknown and should be estimated as the function of the length of the three rods (*ℓ*_*i*_, *i* = 1, 2, 3). We can estimate each rod's total arc length as

(5) $$\ell_{i}(t)=\int_{0}^{l}\|r^{\prime }i(s,t)\|\text{d}s,$$

where ‖⋅‖ denotes the *ℓ*_2_-norm and ***r***_*i*_(*s, t*) is the position of ith rod given by

(6) $$\begin{array}{l} r_{i}\left(s,t\right)=r\left(s,t\right)+\text{R}\left(s,t\right)d_{i}. \end{array}$$

Substituting (6) in (5) and simplifying the equations using (3) yields

(7) $$\ell_{i}(t)=\int_{0}^{l}\|e_{3}+[\text{u}(s,t){]}_{\times }d_{i}\|\text{d}s,$$

Now, we can write the system of differential equations governing the motion of the robot using (3), (4), and (7)

(8a) $$r^{\prime }(s,t)=\text{R}(s,t)e_{3},$$

(8b) $${\text{R}}^{\prime }(s,t)=\text{R}(s,t){\left[\text{u}(s,t)\right]}_{\times },$$

(8c) $$\begin{array}{l} u^{\prime }(s,t)=-K^{-1}[{\left[\text{u}(s,t)\right]}_{\times }Ku(s,t)+\\ \;{\left[e_{3}\right]}_{\times }{\text{R}}^{T}(s,t)(F(t)+(l-s)f)], \end{array}$$

(8d) $$\ell^{\prime }i(s,t)=\|e_{3}+{\left[\text{u}(s,t)\right]}_{\times }d_{i}\|,\;i=1,2,3,$$

with the following boundary conditions

(9a) $$\begin{array}{l} r\left(0,t\right)={\left[0\;0\;0\right]}^{T}, \end{array}$$

(9b) $$\begin{array}{l} \text{R}\left(0,t\right)=\text{I}, \end{array}$$

(9c) $$\begin{array}{l} u_{z}\left(0,t\right)=0, \end{array}$$

(9d) $$\begin{array}{l} \ell_{i}\left(0,t\right)=0, \end{array}$$

(9e) $$\begin{array}{l} \ell_{i}\left(l,t\right)=L_{i}\left(t\right),\;i=1,2. \end{array}$$

The model defined by (8) and (9) accepts the overall length of the first two rods *L*_*i*_, *i* = 1, 2 as inputs and predicts the robot curvature ***u***(*s, t*), position ***r***(*s, t*), and length of the third rod *ℓ*_3_(*t*). We note that the length of the third rod is always defined by the length of the first and second rod.

Additionally, (8) and (9) form a boundary value problem. In the absence of external torques, the initial curvature of the robot along *z* direction is zero (9c). However, the initial curvatures along *x* and *y* directions [i.e., *u*_*x*_(0, *t*) and *u*_*y*_(0, *t*)] are unknown. In addition, the first and second rods' arc length *ℓ*_*i*_(*s, t*), *i* = 1, 2 are defined both at the base (*s* = 0) and the tip of the robot (*s* = *l*) by (9d, 9e).

Moreover, the model given in (8) is quasi-static and solved using the separation of variables. To solve the equations, it is assumed that at a given time, time-dependent variables are constant and the equations are solved in spatial domain (with respect to *s*) using standard methods such as the Runge-Kutta or Adams-Bashforth families of algorithms. Shooting methods can be used to solve the boundary value problem. A shooting method consists of using a nonlinear root-finding algorithm to iteratively converge on values for *u*_*x*_(0, *t*) and *u*_*y*_(0, *t*), in order to satisfy (9e). Next, the time-dependent variables are updated [i.e., *L*_*i*_(*t*)], and the equations are solved again in the spatial domain.

### 2.3. Multi-Segment Robot

**Figure 4:**
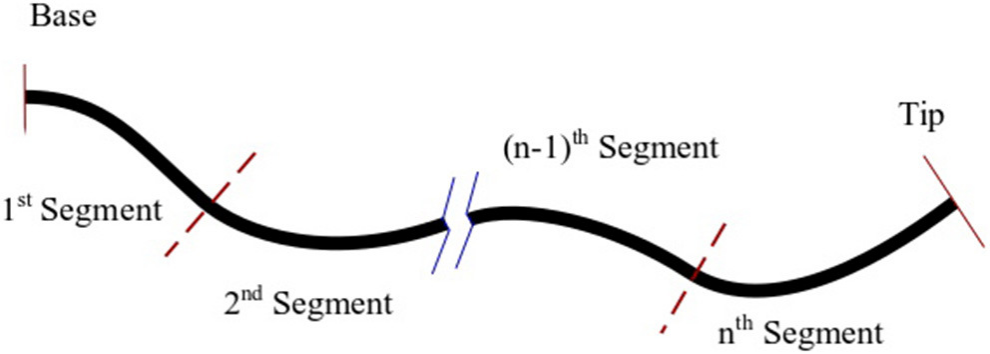
A schematic of multi-backbone robot with multiple bending segments, dashed lines denote break points.

Here, we generalise the model given in (8) for a multi-backbone robot with multiple bending segments shown in Figure 4. It is assumed that the robot is composed of *n* segments with lengths of *l*_*j*_, *j* = 1, .., *n*. Each segment is actuated via 3 parallel rods fixed at the end the segment. Thus, there are *n* rods and the jth segment contains 3 × (*n* + 1 − *j*) rods.

To model the robot, we start from the 1st segment containing *n* × 3 rods and use (8) to estimate the curvature, position of the main backbone, and the lengths of the cables up to the next segment. Next, at the junction where the segment ends (shown with dashed lines in Figure 4) we enforce the appropriate boundary conditions. The boundary conditions to be enforced across each transition point between sections are as follows: (1) The position and orientation of each tube must be continuous across the boundary, i.e.,

(10) $$\begin{array}{l} r\left(s^{-},t\right)=r\left(s^{+},t\right),\text{ R}\left(s^{-},t\right)=\text{R}\left(s^{+},t\right), \end{array}$$

(2) considering the static equilibrium and the fact that the rods apply no torque around *z* direction:

(11) $$\begin{array}{l} u_{z}\left(s^{-},t\right)=u_{z}\left(s^{+},t\right), \end{array}$$

(3) at the distal end of each segment, we have a boundary condition for the length of the rods that end:

(12) $$\begin{array}{l} \ell_{j}\left(s,t\right)=L_{j}\left(t\right). \end{array}$$

We repeat this process for the rest of the segments. We note that the curvatures along *x* and *y* at each break point are unknown. A shooting method must be used to iteratively converge on values for {*u*_*x*_(0, *t*), *u*_*y*_(0, *t*), *u*_*x*_(*l*_1_, *t*), *u*_*y*_(*l*_1_, *t*),…, $u_{x}(\sum_{j=1}^{n}l_{j},t),\;u_{y}(\sum_{j=1}^{n}l_{j},t)\}$, in order to acquire the desired length for the rods. Solving the BVP numerically is computationally intensive. The computational cost of the model is a significant obstacle in deployment of such designs and more efficient numerical methods are needed. To this end, we study the design of a novel observer that can rapidly estimate the solution of robot's model without the need to solve the BVP.

### 2.4. Rapid Solution of the Robot's Model

Our main goal in this section is to design an observer that employs measurements of *ℓ*_*i*_(*l, t*) through time to estimate correct value of *u*_*y*_(0, *t*) and *u*_*x*_(0, *t*) and ensures the boundary conditions in (9) are satisfied without the need to solve the BVP iteratively. First, we transform the model in (8) into an observable form that simplifies the design of the observer. Next, we design an observer rule that guarantees exponential convergence of the solution of the observable model to the solution of the robot model given in by (8) and (9).

We define the vector of missing initial values as

(13) $$\begin{array}{l} \overset{ˇ}{u}\left(0,t\right)={\left[u_{y}\left(0,t\right),\;u_{x}\left(0,t\right)\right]}^{T}. \end{array}$$

To realise the effect of the missing initial value [i.e., $\overset{ˇ}{u}\left(0,t\right)$] on the solution of the equations, we define four auxiliary variables, namely,

(14a) $$\begin{array}{l} A_{i}\left(s,t\right):=\frac{\partial \ell_{i}\left(s,t\right)}{\partial \overset{ˇ}{u}\left(0,t\right)},i=1,2,3 \end{array}$$

(14b) $$\begin{array}{l} B\left(s,t\right):=\frac{\partial u\left(s,t\right)}{\partial \overset{ˇ}{u}\left(0,t\right)}, \end{array}$$

(14c) $$C(s,t):=\frac{\partial \left[{\text{R}}^{T}(s,t)(F(t)+(l-s)f)\right]}{\partial \overset{ˇ}{u}(0,t)},$$

(14d) $$D(s,t):=\frac{\partial \left({\text{R}}^{T}(s,t)f\right)}{\partial \overset{ˇ}{u}(0,t)}.$$

Using the new variables defined by (14), one can derive the following generalised model of the multi-backbone robot (see the Appendix for derivation)

(15a) $$r^{\prime }(s,t)=\text{R}(s,t)e_{3},$$

(15b) $${\text{R}}^{\prime }(s,t)=\text{R}(s,t){\left[\text{u}(s,t)\right]}_{\times },$$

(15c) $$\begin{array}{l} u^{\prime }(s,t)=-K^{-1}[{\left[\text{u}(s,t)\right]}_{\times }Ku(s,t)+\\ \;{\left[e_{3}\right]}_{\times }{\text{R}}^{T}(s,t)(F(t)+(l-s)f)], \end{array}$$

(15d) $${\ell^{\prime }}_{i}(s,t)=\|e_{3}+{\left[\text{u}(s,t)\right]}_{\times }d_{i}\|,\;i=1,2,3,$$

(15e) $${A^{\prime }}_{i}(s,t)=\frac{-{\left(e_{3}+{\left[\text{u}(s,t)\right]}_{\times }d_{i}\right)}^{T}{\left[d_{i}\right]}_{\times }}{\left\|e_{3}+{\left[\text{u}(s,t)\right]}_{\times }d_{i}\right\|}ℬ(s,t),\;i=1,2,3,$$

(15f) $$\begin{array}{l} B^{\prime }(s,t)=K^{-1}[{\left[Ku(s,t)\right]}_{\times }B(s,t)-\\ \;\;{\left[u(s,t)\right]}_{\times }KB(s,t)-{\left[e_{3}\right]}_{\times }C(s,t)], \end{array}$$

(15g) $$\begin{array}{l} C^{\prime }(s,t)=[{\text{R}}^{T}(s,t)(F(t)+(l-s)f){]}_{\times }B(s,t)-\\ \;[u(s,t){]}_{\times }C(s,t)-D(s,t), \end{array}$$

(15h) $$D^{\prime }(s,t)={\left[{\text{R}}^{T}(s,t)f\right]}_{\times }B(s,t)-{\left[u(s,t)\right]}_{\times }D(s,t).$$

Now, we provide a set of initial conditions for (15) that ensures the solution of the observer model in (15) exponentially converges to the solution of the boundary value problem defined by (8) and (9).

(16a) $$\begin{array}{l} r\left(0,t\right)={\left[0\;0\;0\right]}^{T}, \end{array}$$

(16b) $$\begin{array}{l} \text{R}\left(0,t\right)=\text{I}, \end{array}$$

(16c) $$\begin{array}{l} u_{z}\left(0,t\right)=0, \end{array}$$

(16d) $$\begin{array}{l} \left[\begin{array}{c} u_{x}\left(0,t\right)\\ u_{y}\left(0,t\right) \end{array}\right]=-\int_{0}^{t}{\left[\begin{array}{c} A_{1}^{T}\left(l,t\right)\\ A_{2}^{T}\left(l,t\right) \end{array}\right]}^{†}\text{P}\left[\begin{array}{c} \ell_{1}\left(l,t\right)-L_{1}\left(t\right)\\ \ell_{2}\left(l,t\right)-L_{2}\left(t\right) \end{array}\right]\text{d}t, \end{array}$$

(16e) $$\begin{array}{l} \ell_{i}\left(0,t\right)=0, \end{array}$$

(16f) $$\begin{array}{l} A_{i}\left(0,t\right)=0,\;i=1,2, \end{array}$$

(16g) $$\begin{array}{l} B\left(0,t\right)=\left[1\;0\;0;0\;1\;0\right], \end{array}$$

(16h) $$\begin{array}{l} C\left(0,t\right)=0, \end{array}$$

(16i) $$\begin{array}{l} D\left(0,t\right)=0, \end{array}$$

where P is a symmetric positive definite matrix and ^†^ denotes the pseudo-inverse operator. We note that (16f-16i) are calculated based on the definition of the auxiliary variables in (14). (16d) is the main observer rule that guarantees the convergence of the observer (see the Appendix).

The observer given in (15) is quasi-static, similar to the robot's model in (8). However, instead of using an iterative BVP solver, it can be solved as an initial value problem using the initial values given in (16). At a given time *t*, time-dependent variables are assumed constant and the equations are solved in spatial domain. Next, the time-dependent variables are updated [i.e., *L*_*i*_(*t*), *u*_*x*_(0, *t*), *u*_*y*_(0, *t*)]. The updated time-dependant variables are used to solve the equations in the spatial domain again. The observer can be generalised to a multi-segment robot following the approach discussed in section 2.3.

In the next section, series of simulation and experiments are performed to evaluate the model's accuracy and demonstrate the computational efficiency of the observer in comparison with common BVP numerical solvers.

## 3. Results

Simulations and experiments are performed to evaluate the proposed design and modelling theory. The bronchoscopic robot used in the simulations and experiments consists of two bendable segments, shown in Figure 2. The length of the first segment is 500 mm, and the length of the second segment (at the tip) is 40 mm. The outer diameter of the robot is 4.5 mm and the inner diameter of the robot is 2 mm. Twenty-seven circular fixtures each weighting 5 g were equally spaced along the length of the bronchoscope and were rigidly fixed to the main backbone shown in blue in Figure 2.

We performed experiments to identify the developed model parameters and validate the model. First we performed experiments to identify the model parameters. For the identification phase, each rod was commanded to either push or pull the end disks by 5 mm, making the robot to randomly bend to 12 different positions. We estimated the 3D shape of the robot using calibrated stereo rig comprising two Logitech HD Pro C922 webcams. The cameras were running at 1, 080p resolution. As identified through calibration using on average 30 views of a checkerboard, a single pixel corresponded to 0.25 × 0.25 mm on the image plane. Following calibration, the entry point of the robot, i.e., s=0 was estimated in 3D space via triangulation. The robot coordinate frame was aligned to a planar calibration target always visible by the cameras during the experiments.

**Figure 5:**
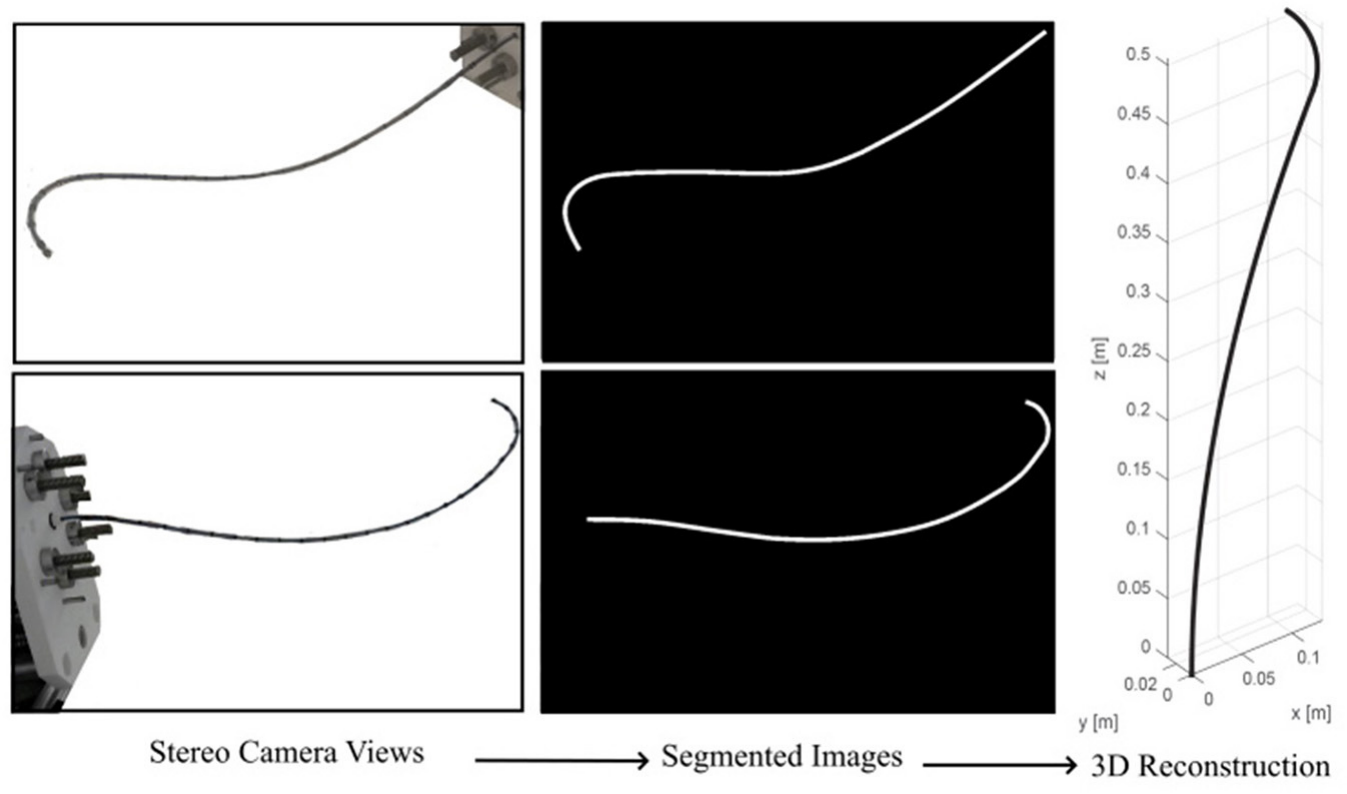
Estimating robot's backbone shape using two calibrated cameras.

Furthermore, manual backbone segmentation established the base and shape of the bronchoscope relative to the aligned calibration grid. Matching backbone points were selected in both images, and then triangulated to provide the 3D point cloud. This process is shown in Figure 5. The mean error of the 3D triangulation algorithm was equal to 6 pixels corresponding to 1.5 mm. The extracted 3D backbones were used to identify for the robot model parameters, namely, Young's modulus, *E* and shear moduli *G* of the robot and initial displacement of the rods, *δℓ*_*i*_, *i* = 1, …, 6. The parameters were identified by fitting the kinematic model given in (8) to the shape of the robot estimated via the cameras at 12 different configurations. The identified parameters of the model and the known parameters of the model are given in Table 1.

**Table 1:** Physical parameters of the robot.

**Known**		**Identified**	
*l*_1_	40 mm	*E*	92.13*e*9 GPa
*l*_2_	500 mm	*G*	31*e*9 GPa
***f***	[0.25, 0, 0]^*T*^ N	*δℓ*_1_	6.03 × 10^−14^ m^4^
*α*_1_	15°	*δℓ*_2_	0.23 mm
*α*_2_	30°	*δℓ*_3_	1.2 mm
*δ*	1.7 mm	*δℓ*_4_	0 mm
*I*	2.13 × 10^−12^ m^4^	*δℓ*_5_	0 mm
*J*	2.72 × 10^−12^ m^4^	*δℓ*_6_	0.7 mm

**Figure 6:**
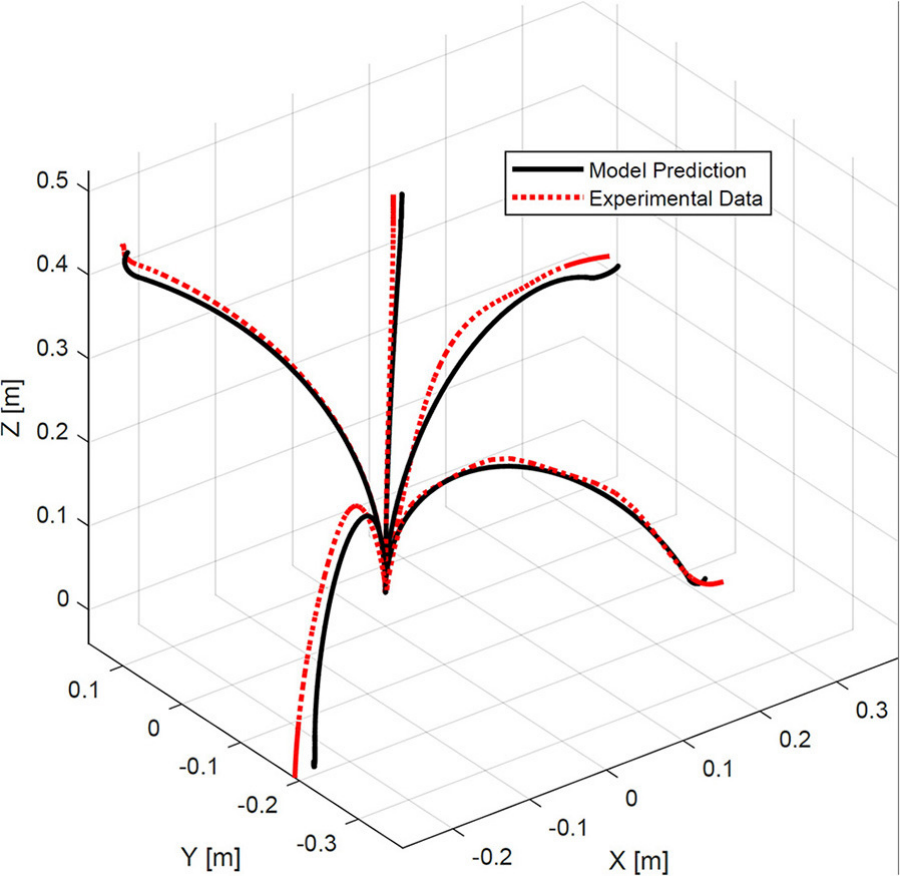
A comparison of experimental bronchoscope's shape with model prediction at four different configurations.

In the next step, to validate the model accuracy we commanded the robot to move to 20 different positions. The shape of the robot was estimated using the calibrated cameras and was compared to the shape of the robot predicted by the identified model. Figure 6 shows representative results. Results of the measurements including maximum, mean, and standard deviation of error of the model in predicting the robot tip position and the root-mean-squared error of the model in predicting robot shape are listed in Table 2. The root-mean-squared error is calculated as

(17) $$\begin{array}{l} \text{RMSE}=\sqrt{\frac{\sum_{j=1}^{m}{\left(\|\hat{r}-r{\|}_{j}\right)}^{2}}{m}}, \end{array}$$

and is used as a measure of the differences between the actual shape of the robot, $\hat{r}$, and the model predicted shape, i.e., ***r***, for *m* = 30 data points along the robot backbone.

**Table 2:** Experimental results.

***e*_*max*_** **[mm]**	***e*_*mean*_** **[mm]**	***σ*** **[mm]**	**RMSE [mm]**
26.2	17.6	10.9	10.3

*Maximum error of tip position (e_max_), mean error of tip position (e_mean_), standard deviation of error (σ), and root mean squared error (RMSE) are reported*.

In the experiments, the robot tip was capable of bending up to 100° with respect to its backbone (see Figure 6). The maximum error of the model in estimating the position of the robot tip is 26.2 mm, corresponding to 1.9% of the robot's length.

Finally, we performed simulations to compare the computational efficiency of the observer with various shooting methods used to solve BVPs. Shooting methods consists of using a nonlinear root-finding algorithm to iteratively converge on values for *u*_*x*_(0, *t*) and *u*_*y*_(0, *t*) for each segment, in order to satisfy the boundary conditions (9), i.e.,

(18) $$\begin{array}{l} \begin{array}{c} \text{Minimize: }Error:=\|\left[\begin{array}{c} \ell_{1}\left(l_{1},t\right)-L_{1}\left(t\right)\\ \ell_{2}\left(l_{1},t\right)-L_{2}\left(t\right)\\ \ell_{4}\left(l_{1}+l_{2},t\right)-L_{4}\left(t\right)\\ \ell_{5}\left(l_{1}+l_{2},t\right)-L_{5}\left(t\right) \end{array}\right]\|,\\ \text{w.r.t.}:\;u_{x}\left(0,t\right),u_{y}\left(0,t\right),u_{x}\left(l_{1},t\right),u_{y}\left(l_{1},t\right). \end{array} \end{array}$$

We compared the observer predictions with shooting method algorithms that employ three different root-finding algorithms, which to the best of authors knowledge, are the most commonly used BVP solvers. These solvers are:

1. Interior-point method (Byrd et al., 2000),
2. Quasi-Newton method with BFGS Hessian estimation (Curtis and Que, 2015),
3. Nelder-Mead method (Powell, 1973).

In the simulations, we pulled and pushed the cables from −5 to 5 mm at a frequency of 2*π*/10 Hz. The simulation runs for 10 seconds at sampling frequency of 50 Hz. The observer gain P used in the simulations was set to 70 × I, as this value was found to achieve the minimum prediction error. The optimally tolerance for all the algorithms were set to 10^−6^. The simulations are performed in Matlab on an Intel Core i7 (2.93 GHz) machine with 16 GB memory.

**Figure 7:**
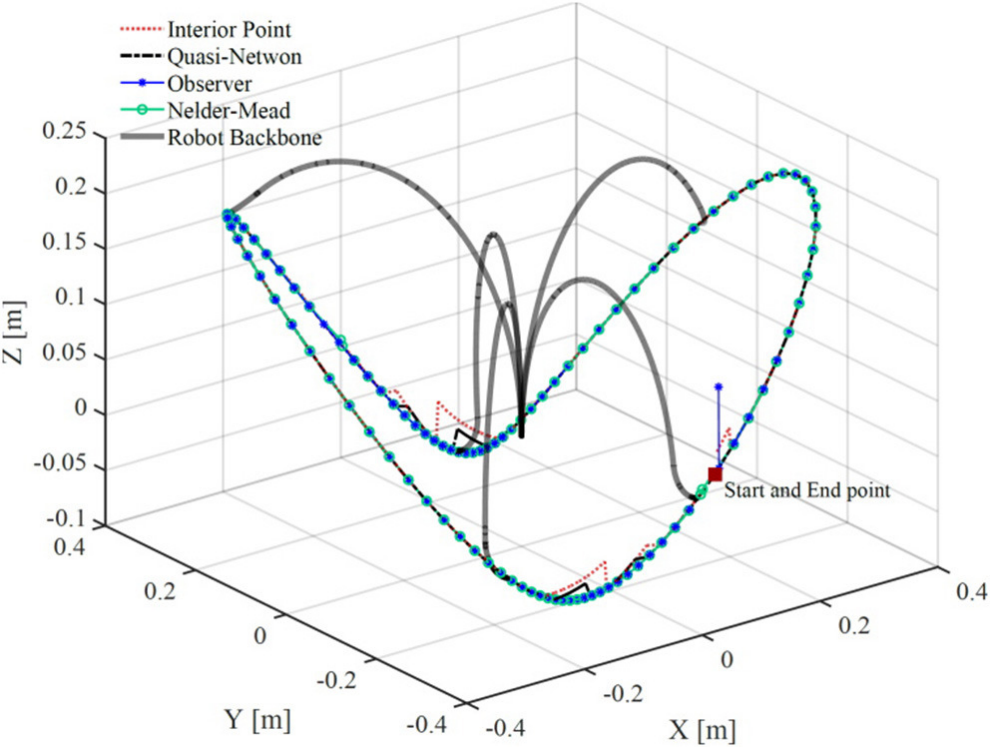
A comparison of bronchoscope's tip trajectory calculated by solving the robot's model using four different methods. The bronchoscope's backbone is shown at several configurations along the trajectory.

Figure 7 shows the robot's trajectory estimated via different methods. The observer and the Nelder-Mead method gave the best accuracy. The other two methods, namely, interior-point and quasi-Newton, gave substantial error at two points across the robot trajectory. Also, it can be seen that the observer has an error at the first sampling time but rapidly converges to the correct solution.

**Figure 8:**
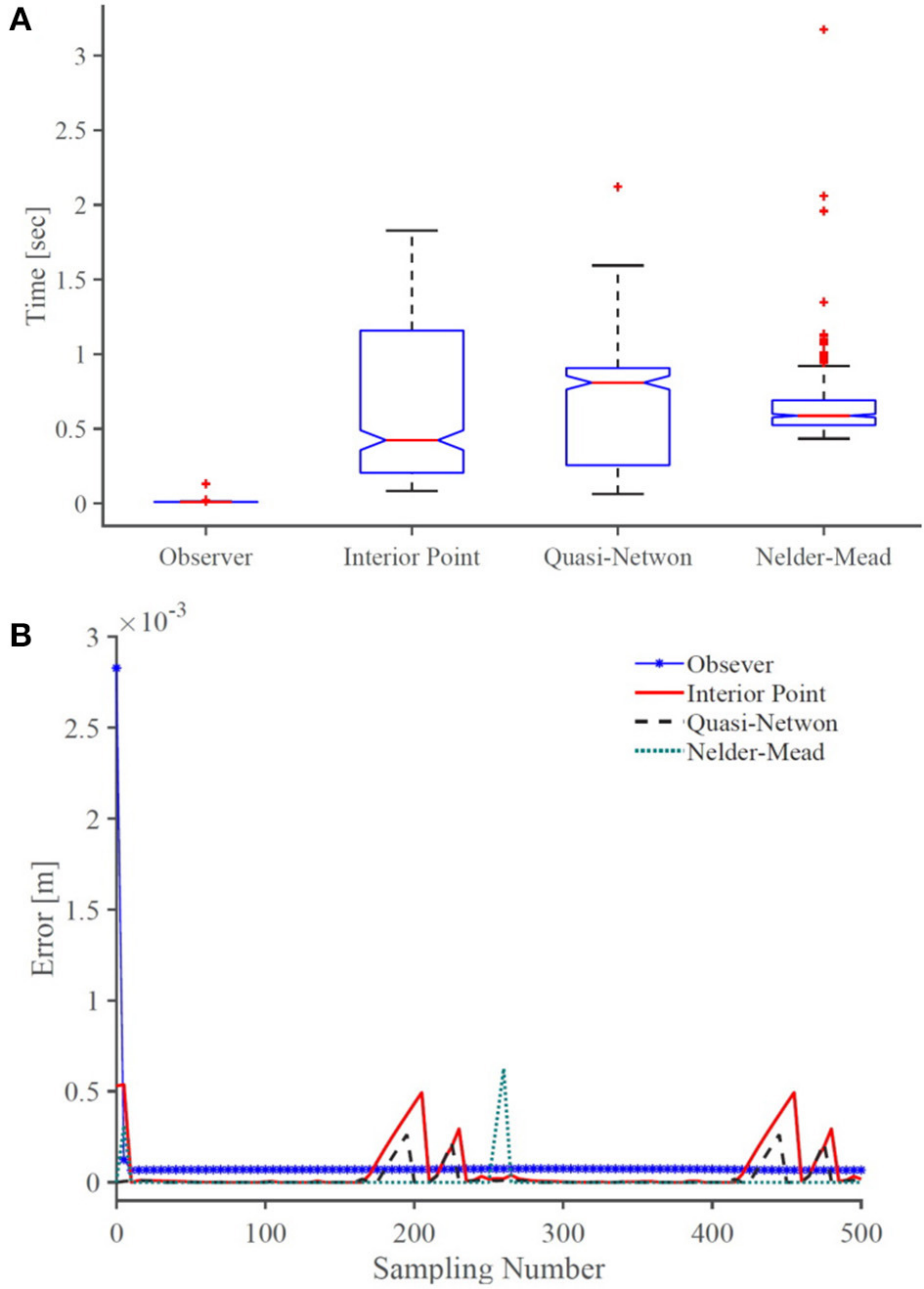
A comparison of **(A)** accuracy and **(B)** computational efficiency of the observer with common BVP solvers. On each box in **(B)**, the central mark indicates the median, and the bottom and top edges of the box indicate the 25th and 75th percentiles, respectively. The whiskers extend to the most extreme data points and the outliers are plotted individually using plus symbol.

Figure 8 shows the error of the solvers and the observer in satisfying the boundary conditions given in (18). The observer error is the same order as the BVP solvers. The BVP solvers occasionally fail in minimizing the error, while the observer consistently maintains an error below 10^−4^ mm. Figure 8 compares the computational efficiency of the BVP solvers and the observer in terms of the time that each method takes to compute the solution of the model at each sampling time. Evidently, the observer is much faster than the BVP solvers and has lower standard deviation. The average time that the observer takes to estimate the model's solution is 0.0108 s, which is significantly faster than other solvers. We performed 10 more simulations, where, the robots rods are pulled/pushed at frequencies between *π*/5 Hz and *π*/50. The results are summarised in Table 3. The results demonstrate that the observer maintain similar error as the BVP solvers, while exhibiting superior computational efficiency. The average time that the observer takes to estimate the model's solution is 47 times faster than the fastest BVP solver, namely, the interior-point method.

**Table 3:** Experimental results.

	**Observer**	**Interior-point**	**Quasi-Newton**	**Nelder-Mead**
*e*_mean_ [mm]	8.05 × 10^−5^	7.28 × 10^−5^	2.53 × 10^−5^	9.52 × 10^−5^
*σ*_*e*_ [mm]	1.35 × 10^−4^	1.39 × 10^−4^	6.14 × 10^−5^	6.04 × 10^−5^
*t*_mean_ [sec]	0.011	0.52	0.62	0.64
*σ*_*t*_ [sec]	0.006	0.45	0.36	0.19

*Mean error (e_mean_), standard deviation of error (σ_e_), average time to estimate the solution of the model (t_mean_) for each method, and standard deviation of time σ_t_ are reported*.

## 4. Discussion

In this paper, we presented the concept for and the design of a continuum robot for pulmonary endoscopy in MV patients. MV patients are at high risk of developing secondary infections and there is a need for a reliable and controlled sampling of the distal lung to guide therapeutic interventions. Current methods for diagnosis of diseases in the distal lung in MV patients are not standardised, lack reproducibility and require expert operators. Here, we proposed a novel robotic bronchoscope that can be used to democratise lung sampling and improve the accuracy and reliability of distal lung sampling in MV patients. The proposed design of the system considers the limitations and constraints of current bronchoscopy, i.e., limited dexterity, low repeatability, and relatively large size of the bronchoscope.

One of the main challenges in current bronchoscopy is navigating the tightly curved architecture of bronchial tree. Several studies (Coppola et al., 1998; Ulusoy et al., 2016) have reported that bifurcation angles of the bronchial tree including sub-carinal angles and inter-bronchial angles vary between 30 and 100°. The experimentally measured maximum bending angle of the proposed robotic bronchoscope is 100° with respect to the robot's main backbone, which enables the robot to traverse the tightly curved structure of airways. We note that the maximum bending angle and curvature of the robot is a function of the robot's interaction with the environment. In the future, we will study the bending capability of the robot in lung models to fully verify the effectiveness of the robot in navigating the bronchial tree.

The external diameter of traditional bronchoscopes is generally 5–6 mm with a working channel with inner diameter of 2 mm (Vachani and Sterman, 2008). The developed prototype is comparable with current technology and has an outer diameter of 4.5 mm with a working channel with inner diameter of 2 mm. Moreover, the bronchoscope is highly dexterous and possesses 7 DoFs in total. The continuum manipulator is able to bend in 3D at 2 different points along its backbone thanks to 6 push/ pull NiTi rods. The extra dexterity offered by the proposed design can potentially extend the reach of the clinical bronchoscopy.

One of the aims of this research is to democratise bronchoscopy in MV patients in the ICU via automating the procedure. To this end, we have proposed a new theoretical framework to model the robot that can be used in closed-loop control of the bronchoscope motion. Our novel mechanics-based model of the robotic bronchoscope can predict the shape of the robotic bronchoscope under external forces with an accuracy corresponding to 1.9% of its arc-length. We note that for long, slender continuum robots, tip error is highly dependent on the total arc length (Rucker et al., 2010) and robot's backbone's interaction with its surrounding environment. We note that this error can be further reduced via closed-loop control of the robot tip. A closed-loop controller can employ sensory feedback from the robot tip position to minimise the bronchoscope error in navigating the lung. In practice, electromagnetic trackers are placed at the tip of the bronchoscope to measure its tip position in real-time. The proposed design offers a 2 mm working channel that can be used to place such trackers, allowing real-time monitoring of robot position for closed-loop control.

Furthermore, we have demonstrated that our numerical framework can estimate the model's solution 47 times faster than the fastest existing solvers, enabling applications in real-time robotic control. Future work will focus on developing a closed-loop control strategy that uses the model and the feedback of the robot tip position acquired with electromagnetic trackers, to minimise the error of the robot tip in following a desired trajectory for sampling.

## Data Availability Statement

The original contributions presented in the study are included in the article/supplementary material, further inquiries can be directed to the corresponding author/s.

## Author Contributions

MK conceived the study. ZM contributed to modelling and design of the robot. BT contributed to the experimental study. All authors contributed to manuscript writing, revision, and read and approved the submitted version.

## Conflict of Interest

The authors declare that the research was conducted in the absence of any commercial or financial relationships that could be construed as a potential conflict of interest.

## Figures and Tables

**Table:** 

*t*	Time.
*s*	Arc length.
^.^	Derivative with respect to time.
^′^	Derivative with respect to *s*.
[ ]__×__	Converts ℝ^3^ in *so*(3).
*n*	Number of bendable segments.
*i*	Number of rods in each segments.
***d***_*i*_	Position of rods with respect to the main backbone.
*δ*	Rods' distance from the robots centroid.
*α*_*n*_	Angular position of 1^st^ rod of *n*^th^ segment with respect to the main backbone.
*β*_*i*_	Angular position of *i*^th^ rod with respect to the main backbone.
*l*	Overall length of the robot.
*l*_*n*_	length of the *n*^th^ segment.
*ℓ*_*i*_	Model predicted length of the *i*^th^ rod.
***r***(*s, t*)	Position vector of the main backbone in the global reference frame.
***r***_*i*_(*s, t*)	Position vector of the *i*^th^ rod in the global reference frame.
R(*s, t*)	Orientation matrix.
***u***(*s, t*)	Vector of backbone curvatures.
*u*_*x*_(*s, t*)	Curvature around *x* axis.
*u*_*y*_(*s, t*)	Curvature around *y* axis.
*u*_*z*_(*s, t*)	Curvature around *z* axis.
Κ	Stiffness matrix.
*E*	Young's modulus.
*I*	Second moment of inertia.
*G*	Shear modulus.
*J*	Polar moment of inertia.
***F***(*t*)	External point force.
***f***	External distributed force.
***e***_3_	Unit vector aligned with the z-axis of the global coordinate frame.
I	Identity matrix.
0	Zero matrix.
P	Observer gain.
A(s,t),B(s,t),C(s,t),D(s,t)	Auxiliary variables defined in (14).

## Appendix

### Derivation of Auxiliary Variables

Here, we derive the differential equations governing the evolution of auxiliary variables given in (14), beginning with (14a).

Using (8d) and the chain rule of differentiation we have

$$\begin{array}{l} A^{\prime }i(s,t)=\\ \frac{\partial }{\overset{ˇ}{u}(0,t)}{\left[{\left(e_{3}+{\left[\text{u}(s,t)\right]}_{\times }d_{i}\right)}^{T}(e_{3}+{\left[\text{u}(s,t)\right]}_{\times }d_{i})\right]}^{1/2}=\\ \frac{-{\left(e_{3}+{\left[\text{u}(s,t)\right]}_{\times }d_{i}\right)}^{T}{\left[d_{i}\right]}_{\times }}{\left\|e_{3}+{\left[\text{u}(s,t)\right]}_{\times }d_{i}\right\|}B(s,t) \end{array}$$

In deriving (15) we used the following identity

$$\frac{\partial ({\left[a\right]}_{\times }b)}{\partial c}=-{\left[b\right]}_{\times }\frac{\partial a}{\partial c}+{\left[a\right]}_{\times }\frac{\partial b}{\partial c}.$$

We now use (8c) to derive the equations for calculating B(s,t).

$$\begin{array}{l} B^{\prime }(s,t)=\frac{\partial u^{\prime }(s,t)}{\overset{ˇ}{u}(0,t)}={\text{K}}^{-1}[{\left[\text{K}u(s,t)\right]}_{\times }B(s,t)-\\ {\;[u(s,t)]}_{\times }\text{K}B(s,t)-{\left[e_{3}\right]}_{\times }C(s,t)]\; \end{array}$$

Furthermore, C(s,t) can be computed by taking the transpose of (8b), multiplying both sides by ***F***(*t*) + (*l* − *s*)*f*, subtracting R^*T*^(*s, t*)***f***, and finally taking partial derivative of both sides with respect to $\overset{ˇ}{u}\left(0,t\right)$.

$$\begin{array}{l} C^{\prime }(s,t)=[{\text{R}}^{T}(s,t)(F(t)+(l-s)f){]}_{\times }B(s,t)-\\ \;[u(s,t){]}_{\times }C(s,t)-D(s,t). \end{array}$$

We can calculate D(s,t) in a similar way. First, we take the transpose of (8b). Next, we multiply both sides by ***f***. Finally, taking partial derivative of both sides with respect to $\overset{ˇ}{u}\left(0,t\right)$ gives

$$D^{\prime }(s,t)={\left[{\text{R}}^{T}(s,t)f\right]}_{\times }ℬ(s,t)-{\left[u(s,t)\right]}_{\times }D(s,t).$$

### Proof of Convergence and Stability

The error of the observer in satisfying the robot's model's boundary condition in (9) (i.e., robot's rods' lengths) is

$$\begin{array}{l} \epsilon \left(t\right)=\left[\begin{array}{c} \ell_{1}\left(l,t\right)\\ \ell_{2}\left(l,t\right) \end{array}\right]-\left[\begin{array}{c} L_{1}\left(t\right)\\ L_{2}\left(t\right). \end{array}\right] \end{array}$$

To prove that this error exponentially converges to zero, we select the following Lyapunov candidate

(A1) $$\begin{array}{l} V=\frac{1}{2}\epsilon^{T}\epsilon . \end{array}$$

Taking the time derivative of *V* and replacing $\dot{\epsilon }$ using (14a) we obtain

$$\dot{V}=\epsilon^{T}\dot{\epsilon }=\epsilon^{T}\left[\begin{array}{c} A_{1}^{T}(l,t)\\ A_{2}^{T}(l,t) \end{array}\right]\;\dot{\overset{ˇ}{u}}(0,t).$$

Substituting $\dot{\overset{ˇ}{u}}\left(0,t\right)$ using (16d) gives

$$\begin{array}{l} \dot{V}=-\epsilon^{T}\left[\begin{array}{c} A_{1}^{T}(l,t)\\ A_{2}^{T}(l,t) \end{array}\right]{\left[\begin{array}{c} A_{1}^{T}(l,t)\\ A_{2}^{T}(l,t) \end{array}\right]}^{†}\text{P}\epsilon \\ \;=-\epsilon^{T}\text{P}\epsilon \end{array}$$

In deriving the above equation we used the following identity

$$\begin{array}{l} \text{a}{\text{a}}^{†}=\text{a}{\text{a}}^{T}{\left(\text{a}{\text{a}}^{T}\right)}^{-1}=\text{I}. \end{array}$$

P is symmetric positive definite. Thus, ∀*t* > 0, $\dot{V}$ is negative definite. Therefore, as *t* → ∞, $\dot{\epsilon }\left(t\right)\to 0$. Consequently, the solution of the observer converges to the solution of the model given in (8).
